# Global epidemiology and species/genotype distribution of *Cryptosporidium* in camels: A systematic review and meta-analysis

**DOI:** 10.1016/j.fawpar.2024.e00235

**Published:** 2024-07-11

**Authors:** Farzad Mahdavi, Farajolah Maleki, Mohammad Reza Mohammadi, Ali Asghari, Behnam Mohammadi-Ghalehbin

**Affiliations:** a Department of Medical Parasitology and Mycology, School of Medicine, Zanjan University of Medical Sciences, Zanjan, Iran; b Clinical Research Development Unit, Shahid Mostafa Khomeini Hospital, Ilam University of Medical Sciences, Ilam, Iran; c Department of Bacteriology, Faculty of Medical Sciences, Tarbiat Modares University, Tehran, Iran; d Social Determinants of Health Research Center, Research Institute for Prevention of Non-Communicable Diseases, Qazvin University of Medical Sciences, Qazvin, Iran; e Zoonoses Research Center, Ardabil University of Medical Sciences, Ardabil, Iran

**Keywords:** *Cryptosporidium* spp., Prevalence, Genotypes, Camels, Meta-analysis

## Abstract

This review analyzed reported data of *Cryptosporidium* prevalence in camels and the species/genotype distribution. Four databases (PubMed, Web of Science, Scopus, Google Scholar) were screened, and studies published by April 1, 2024, were included. Total estimates and 95% CIs were calculated using a random-effects model. The weighted prevalence of *Cryptosporidium* spp. in 7372 camels examined from 12 different countries was estimated at 13.8% with a 95% CI of 10.3–18.4%. The sensitivity analysis based on excluding the individual studies did not result in significant statistical changes in the final weighted prevalence. Subgroup prevalence of *Cryptosporidium* spp. in camels was analyzed by publication year, continent, WHO region, country, camel type, sample size, diagnostic method, age, and gender. A significant publication bias (*P* < 0.05) was reported in the present study. Limitations encountered in this study encompassed: insufficient study diversity, reliance on single study results, inadequate molecular and serological studies in comparison to microscopic studies, etc., all of which could impact the findings. The study identified eight *Cryptosporidium* spp. in camels: *C. parvum*, *C. andersoni*, *C. bovis*, *C. muris*, *C. ratti*, *C. occultus*, *C. ubiquitum*, and *C. hominis*. The first three species had pooled prevalence rates of 65.5%, 66%, and 19.2%, respectively. Each of the remaining five species was documented using a single dataset/study. Moreover, genotypes IIdA19G1, IIaA15G1R1, If-like-A15G2, IIdA15G1, IIaA15G2R1, IIaA17G2R1, and IIaA18G2R1 (*C. parvum*), genotype IV (*C. ratti*), genotype XIIa (*C. ubiquitum*), and genotype IkA19G1 (*C. hominis*) have been identified in camels globally. The findings suggest that camels can act as a source of infection for a variety of *Cryptosporidium* species/genotypes, and can therefore play a key role in disseminating this protozoan to humans and animals.

## Highlights

• The prevalence of *Cryptosporidium* spp. in camels was estimated at 13.8% with a 95% CI of 10.3–18.4%.
• Eight species of *Cryptosporidium* have been identified in camels: *C. parvum*, *C. andersoni*, *C. bovis*, *C. muris*, *C. ratti*, *C. occultus*, *C. ubiquitum*, and *C. hominis*.
• Camels can transmit zoonotic *Cryptosporidium* to humans and animals.

## 1 Introduction

With over 120 genotypes and 44 valid species, *Cryptosporidium* is a major public health concern due to its zoonotic nature (Ryan et al., 2021). In 2004, cryptosporidiosis was included in the World Health Organization's “Neglected Diseases Initiative,” which covers diseases primarily affecting people in low-resource settings (Savioli et al., 2006). The association between immunocompromised individuals (AIDS/HIV) and cryptosporidiosis instances elevated *Cryptosporidium* to a prominent position as a common human infection. In an immunocompetent person, *Cryptosporidium* infection may not show any symptoms or may result in a transient diarrhea. However, *Cryptosporidium* can result in severe, persistent, and sometimes fatal diarrhea as well as acute malnourishment or wasting in immunocompromised people (Izadi et al., 2012; Utami et al., 2020).

In neonatal animals, cryptosporidiosis results in severe diarrhea. However, adult animals continue to be the primary source of infection and are typically asymptomatic carriers (Mosier and Oberst, 2000; Zhang et al., 2022). In recent years, there has been increased recognition of the role of camels as source of infection for *Cryptosporidium* spp., prompting a growing interest in understanding the prevalence, species/genotype distribution, and zoonotic potential of these parasites in camel populations. Given the close interaction between camels and humans in various parts of the world, the zoonotic potential of *Cryptosporidium* spp. in camels has significant implications for public health. Therefore, a comprehensive assessment of the global prevalence and species/genotype diversity of *Cryptosporidium* spp. in camels is essential for understanding the epidemiology of camel-associated cryptosporidiosis and for informing public health and veterinary interventions. This systematic review and meta-analysis aim to synthesize the available evidence on the prevalence and species/genotype distribution of *Cryptosporidium* spp. in camels, providing valuable insights into the global epidemiology of camel-associated cryptosporidiosis and its implications for zoonotic transmission.

## 2 Methods

### 2.1 Ethics approval

The present study was approved by the Ethics Committee of Ardabil University of Medical Sciences, Ardabil, Iran (approval no. IR. ARUMS.REC.1402.386).

### 2.2 Search strategy

In this study, the design, reporting, and interpretation of the data collected from published literature were conducted following the standard protocol of the “Preferred Reporting Items for Systematic Reviews and Meta-Analyses” (PRISMA) checklist (Moher et al., 2015). Peer-reviewed published papers and abstracts on the prevalence of *Cryptosporidium* spp. in camels were identified through systematic searches in four international electronic databases (PubMed, Web of Science, Scopus, and Google Scholar). This search was conducted by two analysts, independently, without any time restrictions up to April 1, 2024. The search was conducted using Medical Subject Heading (MeSH) terms alone or in combination: (“Intestinal Parasites” OR “Parasitic Infections” OR “*Cryptosporidium*” OR “*Cryptosporidium* spp.” OR “Cryptosporidiosis”) AND (“Prevalence” OR “Epidemiology” OR “Frequency” OR “Occurrence”) AND (“Subtype” OR “Genotype” OR “Genotyping”) AND (“Ungulates” OR “Camelids” OR “Camels” OR “Animals”). Additionally, the bibliographies of the original and review articles were thoroughly examined to identify other potential articles that were not retrieved during the database search.

### 2.3 Eligibility criteria, study selection, and data extraction

The eligibility evaluation process proceeded as follows: 1) initial screening using title and abstract, 2) eliminating duplicate records, 3) acquiring full text of relevant papers, and final eligibility verification. Subsequently, three analysts extracted essential information for the meta-analysis stage, which was then validated by two other analysts. Any discrepancies or disagreements were resolved through consensus and discussion with the project's principal investigator. The inclusion criteria for this study were: (1) the study population was restricted to camels, (2) all cross-sectional and epidemiological studies without language or geographical restrictions, (3) studies investigating *Cryptosporidium* spp. in camel feces using molecular, microscopic, and/or serological detection methods, (4) studies published until April 1, 2024, and (5) reporting total sample size and prevalence rates for *Cryptosporidium* spp. Articles that did not mention the prevalence of *Cryptosporidium* spp. in camels, studies on *Cryptosporidium* spp. in non-camel species, research on tissue and blood samples, experimental infections in camels, case studies, reviews, letters, and articles with unclear information were excluded from this study. Variables extracted from each record included: the first author's last name, study implementation time, publication year, WHO regions, countries where the study was done, camel types, age groups, genders, diagnostic methods, total sample sizes, infected samples, and *Cryptosporidium* prevalence rates. Using molecular data, we also assessed the global distribution of different *Cryptosporidium* species and genotypes isolated from camels.

### 2.4 Quality assessment

The quality of the articles was evaluated using the “Joanna Briggs Institute (JBI) critical appraisal checklist” (Institute, J.B, 2017). Papers scoring 4–6 and > 6 points were deemed moderately and highly qualified, respectively. Articles with ≤3 points were excluded from the systematic review.

### 2.5 Data synthesis and meta-analysis

In this study, all statistical analyses were performed using the Comprehensive Meta-Analysis (CMA) v3 software. *P*-values <0.05 were deemed statistically significant. The random-effects model was utilized to evaluate the prevalence of *Cryptosporidium* spp. in camels by estimating pooled prevalence and 95% CIs (Asghari et al., 2023). Sub-group analysis was conducted to assess the weighted prevalence of infection in camels according to camel types, WHO regions, countries, publication years, continents, sample size, diagnostic methods, genders, and age groups. A forest plot diagram was created to display the pooled prevalence with 95% CIs. The funnel plot was used to assess publication bias in the analysis. Heterogeneity among studies was evaluated using the I^2^ index, with values below 25%, 25–50%, and over 50% considered as low, moderate, and high heterogeneity, respectively (Mahdavi et al., 2021). Furthermore, sensitivity analysis was conducted to evaluate variations in the final weighted prevalence of *Cryptosporidium* infection after excluding individual studies.

## 3 Results

### 3.1 Study selection

**Fig. 1:**
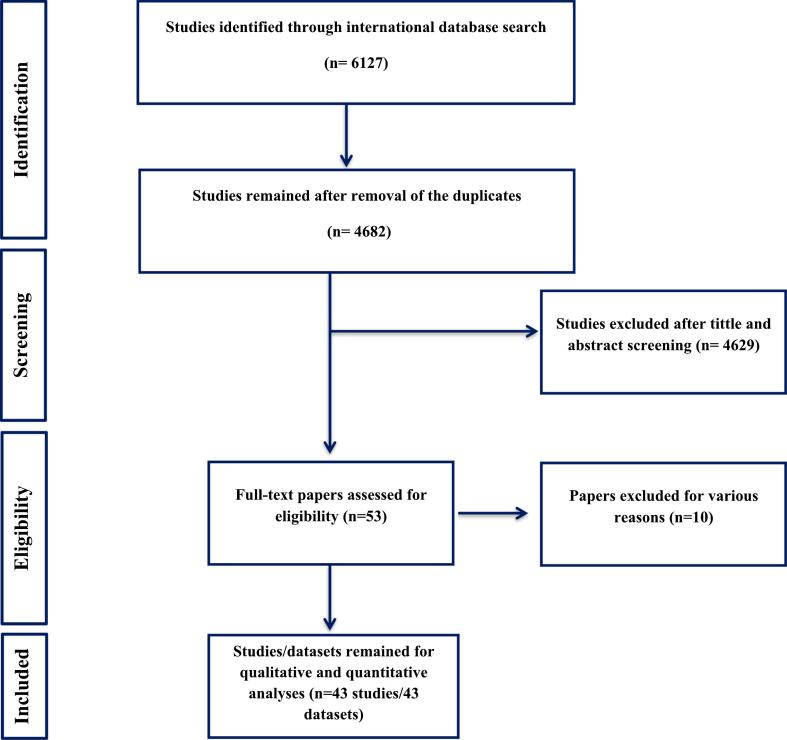
Flowchart depicting the process of included studies in the present review.

The four searched global databases yielded a total of 6127 initial records. After eliminating duplicates and conducting a final review of the remaining 4682 records, 53 articles were ultimately included. Additionally, a quality evaluation based on JBI criteria led to the exclusion of six more studies. Finally, 43 highly qualified papers with 43 datasets met the criteria for inclusion in the present study (Fig. 1).

### 3.2 Qualitative and quantitative characteristics of included papers

The key features of the articles included are outlined in Table 1. These studies span from 1996 to 2023 and analyzed 7372 camel fecal samples globally. A total of 25 studies/datasets were related to one-humped camels (*Camelus dromedarius*), five datasets were related to two-humped camels (*Camelus bactrianus*), and 13 datasets were related to unknown camels (Camel spp.). Geographically, the research was distributed as follows: 10 studies in Egypt, six in Iran, six in Iraq, six in Algeria, five in China, three in Saudi Arabia, two in Ethiopia, one in Azerbaijan, one in Kuwait, one in Nigeria, one in Tunisia, and one in the USA. The sample size ranged from 4 to 1097 camels examined. Out of 43 studies/datasets on *Cryptosporidium* infection in camels, 12 papers (12 datasets) detailed the species/genotype distribution of this parasite. Among diagnostic methods, microscopy was predominantly used in most studies (27 datasets), with molecular and serological techniques accounting for 12 and four datasets, respectively. A total of 11 datasets mentioned the age group of the studied camels, while nine datasets specified the gender. The JBI checklist showed that 22 papers (22 datasets) had high quality (>6 points), while the other 21 articles (21 datasets) had moderate quality (4–6 points) (Supplementary Table 1).

### 3.3 Global prevalence of *Cryptosporidium* spp. infection in camels

**Fig. 2:**
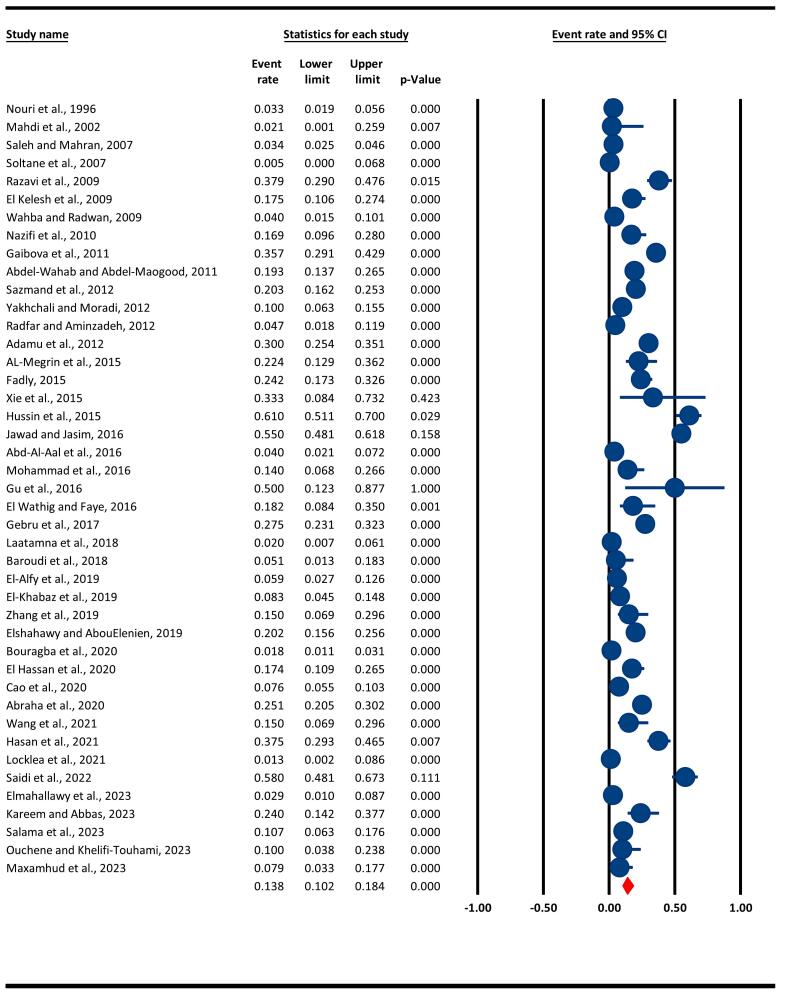
The overall prevalence of *Cryptosporidium* spp. in camels, based on data from the included studies, using a random-effects model and 95% confidence intervals. * Blue colors indicate the event rate/prevalence reported in each study, while the red color represents the final weighted prevalence. (For interpretation of the references to color in this figure legend, the reader is referred to the web version of this article.)

The overall frequency of *Cryptosporidium* spp. infection in camels was estimated at 13.8% with a 95% CI of 10.3–18.4% (Fig. 2). Heterogeneity analysis indicated significant level of heterogeneity in this meta-analysis (Q = 854.1, I^2^ = 95.1%, *P* = 0.000).

**Table 1:** The main details of 43 articles about the occurrence of *Cryptosporidium* spp. in camels. These articles were screened as highly relevant and data extracted from them were used in this study and listed in the table.

Data source	Animal scientific names	Time tested	Countries	Total samples (no.)	Infected samples (no.)	Prevalence (%)	Diagnostic method
Nouri et al., 1996	*Camelus dromedarius*	UC^a^	Iran	396	13	3.3	Mic (Ziehl-Neelsen)^b^
Mahdi and Ali, 2002	*Camel* spp.	UC	Iraq	23	0	0	Mic (Ziehl-Neelsen)
Saleh and Mahran, 2007	*Camelus dromedarius*	2005–2006	Egypt	1097	37	3.4	Mic (Ziehl-Neelsen)
Soltane et al., 2007	*Camel* spp.	2003–2004	Tunisia	110	0	0	Mic (Ziehl-Neelsen)
Razavi et al., 2009	*Camelus dromedarius*	UC	Iran	103	39	37.9	Mic (Ziehl-Neelsen)
El Kelesh et al., 2009	*Camelus dromedarius*	UC	Egypt	80	14	17.5	Mic (Ziehl-Neelsen)
Wahba and Radwan, 2009	*Camelus dromedarius*	UC	Egypt	101	4	3.8	Mic (Ziehl-Neelsen)
Nazifi et al., 2010	*Camelus dromedarius*	2008	Iran	65	11	6.9	Mic (Ziehl-Neelsen)
Gaibova et al., 2011	*Camelus bactrianus*	UC	Azerbaijan	182	65	35.7	Mic (Ziehl-Neelsen)
Abdel-Wahab and Abdel-Maogood, 2011	*Camelus dromedarius*	UC	Egypt	145	28	19.3	PCR^c^
Sazmand et al., 2012	*Camel* spp.	2008–2010	Iran	300	61	20.3	Mic (Ziehl-Neelsen)
Yakhchali and Moradi, 2012	*Camelus dromedarius*	2009–2010	Iran	170	17	10	Mic (Ziehl-Neelsen)
Radfar and Aminzadeh, 2012	*Camelus dromedarius*	UC	Iran	85	4	4.7	ELISA^d^
Adamu et al., 2012	*Camelus dromedarius*	UC	Nigeria	340	102	30	Mic (Ziehl-Neelsen)
Al-Megrin, 2015	*Camel* spp.	2014–2015	Saudi Arabia	49	11	22.4	ELISA
Fadly, 2015	*Camel* spp.	2014–2015	Egypt	120	29	24.2	Mic (Ziehl-Neelsen)
Xie et al., 2015	*Camelus bactrianus*	UC	China	6	2	33.3	PCR and MLST^e^
Hussin et al., 2015	*Camel* spp.	2014–2015	Iraq	100	61	61	Mic (Ziehl-Neelsen)
Jawad and Jasim, 2016	*Camelus dromedarius*	2015–2016	Iraq	200	110	55	Mic (Ziehl-Neelsen)
Abd-Al-Aal et al., 2016	*Camel* spp.	2014–2015	Kuwait	253	10	3.9	Mic (Ziehl-Neelsen) and IC^f^
Mohammed et al., 2016	*Camel* spp.	UC	Iraq	50	7	14	PCR
Gu et al., 2016	*Camelus dromedarius*	UC	China	4	2	50	PCR-RFLP^g^
El Wathig and Faye, 2016	*Camelus dromedarius*	UC	Saudi Arabia	33	6	15.1	Mic (Ziehl-Neelsen)
Gebru et al., 2017	*Camel* spp.	2013–2014	Ethiopia	357	98	27.4	Mic (Ziehl-Neelsen)
Laatamna et al., 2018	*Camelus dromedarius*	UC	Algeria	149	3	2	Mic (Ziehl-Neelsen)
Baroudi et al., 2018	*Camelus dromedarius*	2012–2013	Algeria	39	2	5.1	PCR-RFLP
El-Alfy et al., 2019	*Camelus dromedarius*	2017–2018	Egypt	101	6	5.9	PCR-RFLP
El-Khabaz et al., 2019	*Camelus dromedarius*	UC	Egypt	120	10	8.3	Mic (Ziehl-Neelsen)
Zhang et al., 2019	*Camel* spp.	2018	China	40	6	15	PCR
Elshahawy and AbouElenien, 2019	*Camelus dromedarius*	2016–2017	Egypt	248	50	20.2	Mic (Ziehl-Neelsen)
Bouragba et al., 2020	*Camelus dromedarius*	2015–2018	Algeria	717	13	1.8	Mic (Ziehl-Neelsen)
El Hassan et al., 2020	*Camelus dromedarius*	UC	Saudi Arabia	92	16	17.4	ELISA
Cao et al., 2020	*Camelus bactrianus*	2016–2019	China	476	36	7.6	PCR
Abraha et al., 2020	*Camelus dromedarius*	UC	Ethiopia	307	77	25.1	Mic (Ziehl-Neelsen)
Wang et al., 2021	*Camelus bactrianus*	UC	China	40	6	15	Mic (Ziehl-Neelsen)
Hasan et al., 2021	*Camel* spp.	UC	Iraq	120	45	37.5	Mic (Ziehl-Neelsen)
Locklear et al., 2021	*Camel* spp.	UC	USA	77	1	1.3	Mic (Ziehl-Neelsen)
Saidi et al., 2022	*Camelus dromedarius*	2019	Algeria	100	58	58	Mic (Ziehl-Neelsen)
Elmahallawy et al., 2023	*Camelus bactrianus*	2021	Egypt	102	3	2.9	PCR
Kareem and Abbas, 2023	*Camel* spp.	2022	Iraq	50	12	24	Real time-PCR
Salama et al., 2023	*Camelus dromedarius*	2020–2021	Egypt	121	13	10.7	PCR
Ouchene and Khelifi-Touhami, 2023	*Camelus dromedarius*	2011	Algeria	40	4	10	IFA^h^
Maxamhud et al., 2023	*Camelus dromedarius*	UC	Algeria	63	5	7	PCR

a UC: Unclear.

b Microscopic detection method and Ziehl-Neelsen staining.

c Polymerase Chain Reaction.

d Enzyme-Linked Immunosorbent Assay.

e Multilocus Sequence Typing.

f Immunochromatographic assay.

g Restriction Fragment Length Polymorphism.

h Indirect Fluorescent Antibody.

### 3.4 Weighted prevalence of *Cryptosporidium* spp. infection in camels based on examined subgroups

The subgroup-based prevalence of cryptosporidiosis in camels is shown in Table 2 (Supplementary Fig. 1, Supplementary Fig. 2, Supplementary Fig. 3, Supplementary Fig. 4, Supplementary Fig. 5, Supplementary Fig. 6, Supplementary Fig. 7, Supplementary Fig. 8, Supplementary Fig. 9). In brief, the most common occurrence of *Cryptosporidium* spp. in camels was noted in studies published from 2009 to 2015 (21.7%; 95% CI: 15.7–29.1%). Furthermore, camels in Europe (35.7%; 95% CI: 29.1–42.9%), the EUR WHO region (35.7%; 95% CI: 29.1–42.9%), and Azerbaijan (35.7%; 95% CI: 29.1–42.9%) all exhibited the highest incidence of *Cryptosporidium* spp. with only one study conducted for each. A direct correlation was observed between an increase in sample size and a lower prevalence of *Cryptosporidium* spp. in camels. Uncategorized camels/camel spp. (17.8%; 95% CI: 11.5–26.5%), camels under 5 years old (13.6%; 95% CI: 6.6–26.3%), and male camels (14.1%; 95% CI: 6.8–26.9%) exhibited the highest prevalence of *Cryptosporidium* spp. Moreover, articles that reported *Cryptosporidium* spp. prevalence among camels indicated a higher occurrence of this protozoan when using microscopic (14.8%; 95% CI: 10.1–21.1%) compared to serological (13%; 95% CI: 7–22.9%) and molecular (11.8%; 95% CI: 8–17%) methods.

### 3.5 Sensitivity analysis

After performing the sensitivity analysis, removing specific camel-related datasets did not report any significant changes in the final frequency (Supplementary Fig. 10).

### 3.6 Global distribution of *Cryptosporidium* species in camels

Based on 12 molecular/serological studies in Iran, Egypt, China, Algeria, and Saudi Arabia, seven datasets [65.5% (95% CI: 30.5–89.2%)], four datasets [66% (95% CI: 51.7–77.9%), and four datasets [19.2% (95% CI: 4.6–54.1%)] reported *C. parvum*, *C. andersoni*, and *C. bovis* in camels, respectively. From three datasets, 79.6% (95% CI: 33.8–96.7%) of samples remained unidentified as *Cryptosporidium* spp. (Supplementary Fig. 11). Each of *C. muris*, *C. ratti*, *C. occultus*, *C. ubiquitum*, and *C. hominis* species was found in only one dataset (Table 3).

### 3.7 Global distribution of *Cryptosporidium* genotypes in camels

From three datasets, *C. parvum* genotypes IIdA19G1, IIaA15G1R1, If-like-A15G2, IIdA15G1, IIaA15G2R1, IIaA17G2R1, and IIaA18G2R1 have been documented in Egypt, China, and Algeria. In Egypt, genotype IV of *C. ratti* has been identified. In China, genotypes XIIa and IkA19G1 have been found in *C. ubiquitum* and *C. hominis*, respectively (Table 3).

**Table 2:** Subgroup analysis of *Cryptosporidium* spp. in examined camels according to publication year, continent, WHO region, country, camel type, sample size, diagnostic method, age, and gender.

Subgroup variable	Prevalence % (95% CI)	Heterogeneity (Q)	df (Q)	I^2^ (%)	p-value
Publication year					
<2000	3.3 (1.9–5.6)	0	0	0	p > 0.05
2002–2008	3 (1.7–5.4)	2.1	2	7.2	*p* > 0.05
2009–2015	21.7 (15.7–29.1)	138.9	13	90.6	p < 0.05
2016–2022	13.9 (8.7–21.3)	438.7	19	95.7	*p* < 0.05
>2022	10 (5.3–18.2)	14.3	4	72	p > 0.05
Continent					
Africa	10.5 (6.7–16.1)	423.4	19	95.5	p > 0.05
Asia	17.9 (11.5–26.8)	384.4	20	94.8	p > 0.05
Europe	35.7 (29.1–42.9)	0	0	0	p > 0.05
North America	1.3 (0.2–8.6)	0	0	0	p > 0.05
WHO region					
AFR	13.2 (7–23.4)	203.5	8	96.1	p < 0.05
AMR	1.3 (0.2–8.6)	0	0	0	p > 0.05
EMR	13.4 (8.9–19.6)	552.4	26	95.3	p < 0.05
EUR	35.7 (29.1–42.9)	0	0	0	p > 0.05
WPR	15.9 (8.2–28.6)	13.1	4	69.5	p < 0.05
Country					
Algeria	7.6 (1.2–35)	186.3	5	97.3	p < 0.05
Azerbaijan	35.7 (29.1–42.9)	0	0	0	p > 0.05
China	15.9 (8.2–28.6)	13.1	4	69.5	p < 0.05
Egypt	9.7 (5.6–16.4)	123.7	9	92.7	p < 0.05
Ethiopia	26.4 (23.2–29.9)	0.5	1	0	p > 0.05
Iran	12.3 (5.8–24.1)	85.2	5	94.1	p < 0.05
Iraq	34.2 (20.9–50.5)	50.8	5	90.1	p < 0.05
Kuwait	4 (2.1–7.2)	0	0	0	p > 0.05
Nigeria	30 (25.4–35.1)	0	0	0	p > 0.05
Saudi Arabia	19.1 (13.9–25.6)	0.5	2	0	p > 0.05
Tunisia	0.5 (0–6.8)	0	0	0	p > 0.05
USA	1.3 (0.2–8.6)	0	0	0	p > 0.05
Sample size					
<100	14.4 (10.9–18.6)	31.6	16	49.4	p < 0.05
100–300	16.6 (10.9–24.5)	387.9	18	95.4	p < 0.05
301–500	15.1 (7.9–26.9)	127.5	4	96.9	p < 0.05
>500	2.6 (1.4–4.7)	3.8	1	73.8	p > 0.05
Diagnostic method					
Mic	14.8 (10.1–21.1)	729.2	26	96.4	p < 0.05
Mol	11.8 (8–17)	41.5	11	73.5	p < 0.05
Sero^a^	13 (7–22.9)	9.4	3	68.3	p < 0.05
Camel type					
BC^b^	14 (4.8–34.2)	81	4	95.1	p < 0.05
Camel spp.	17.8 (11.5–26.5)	141.2	12	91.5	p < 0.05
DC^c^	12.6 (8.1–19)	608.3	24	96.5	p < 0.05
Age groups (y)					
<5	13.6 (6.6–26.3)	201.6	10	95	p < 0.05
5–10	11.8 (5.7–22.9)	34.3	7	79.6	p < 0.05
>10	7.1 (0.1–85.7)	9.3	1	89.2	p < 0.05
Gender					
Female	12.3 (4.3–30.4)	147.9	8	94.6	p < 0.05
Male	14.1 (6.8–26.9)	80.8	8	90.1	p < 0.05

a Serological detection method.

b Bactrian camel.

c Dromedary camel.

**Table 3:** Summary of the reported data on *Cryptosporidium* species and genotypes in camels.

Data source	Total samples (no.)	Infected samples (no.)	Countries	Camel types	Species identified (genotypes): % (infected no./total no.)
Abdel-Wahab and Abdel-Maogood, 2011	145	28	Egypt	*Camelus dromedarius*	*C. muris* (UC)
Radfar and Aminzadeh, 2012	85	4	Iran	*Camelus dromedarius*	*C. parvum*: 50 (2/2), *Cryptosporidium* spp.: 50 (2/2)
Xie et al., 2015	6	2	China	*Camelus bactrianus*	*C. andersoni*: 100 (2/2)
Gu et al., 2016	4	2	China	*Camelus dromedarius*	*C. andersoni*: 50 (2/4)
Baroudi et al., 2018	39	2	Algeria	*Camelus dromedarius*	*C. parvum*: 100 (2/2)
El-Alfy et al., 2019	101	6	Egypt	*Camelus dromedaries*	*C. parvum* (IIdA19G1- IIaA15G1R1): 33.3 (2/6), *C. ratti* (genotype IV): 16.7 (1/6), and *Cryptosporidium* spp.: 50 (3/6)
Zhang et al., 2019	40	6	China	Camel spp.	*C. andersoni*: 66.7 (4/6), *C. bovis*: 33.3 (2/6)
El Hassan et al., 2020	92	16	Saudi Arabia	*Camelus dromedarius*	*C. parvum*: 100 (16/16)
Cao et al., 2020	476	36	China	*Camelus bactrianus*	*C. andersoni*: 66.7 (24/36), *C. parvum* (If-like-A15G2 and IIdA15G1): 16.7 (6/36), *C. occultus* 5.5 (2/36), *C. ubiquitum* (XIIa): 5.5 (2/36), *C. hominis* (IkA19G1): 2.8 (1/36), and *C. bovis*: 2.8 (1/36)
Elmahallawy et al., 2023	102	3	Egypt	*Camelus bactrianus*	*C. bovis*: 33.3 (1/3), *C. parvum*: 66.7 (2/3)
Salama et al., 2023	121	13	Egypt	*Camelus dromedarius*	*Cryptosporidium* spp.: 100 (13/13)
Maxamhud et al., 2023	63	5	Algeria	*Camelus dromedarius*	*C. parvum* (IIaA15G2R1, IIaA17G2R1, IIaA18G2R1, and IIdA19G1): 80 (4/5), *C. bovis*: 20 (1/5)

### 3.8 Publication bias

**Fig. 3:**
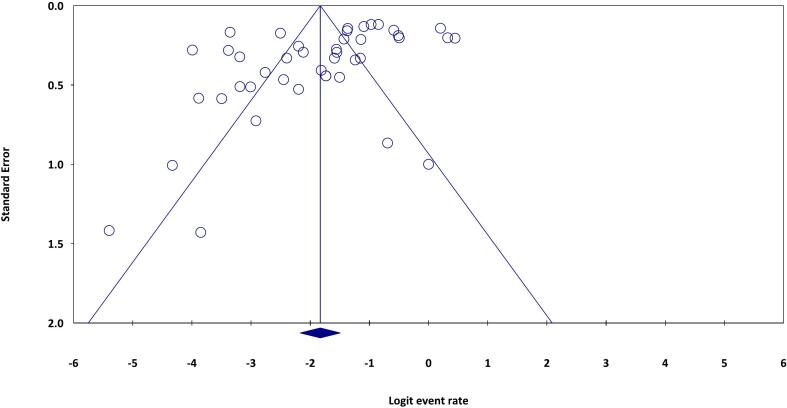
The funnel plot shows the publication bias in the present study.

A substantial publication bias was identified in the present systematic review and meta-analysis (Egger's regression: intercept = − 3.765, 95% lower limit = − 6.325, 95% upper limit = − 1.204, t-value = 2.97, *P* = 0.004) (Fig. 3).

## 4 Discussion

Evaluating *Cryptosporidium* infection in animals, particularly camels, is significant for several reasons. Firstly, *Cryptosporidium* is a parasite that can cause gastrointestinal illness in animals, leading to symptoms such as diarrhea, dehydration, and weight loss. By identifying and monitoring *Cryptosporidium* infection in animals, veterinarians and researchers can better understand the prevalence and impact of the parasite in different populations. Additionally, cryptosporidiosis is a zoonotic disease, meaning it can be transmitted from animals to humans. Camels are commonly used for milk production and as working animals in certain regions, so evaluating *Cryptosporidium* infection in camels is important for assessing the potential risk of transmission to humans. This information can help inform public health measures to prevent and control the spread of the parasite (Saleh and Mahran, 2007; Sazmand et al., 2012; Wang et al., 2021).

There has been no comprehensive review focusing on the prevalence, species/genotypes distribution, and zoonotic importance of *Cryptosporidium* in camels. Non-animal meta-analyses have indicated a global prevalence of *Cryptosporidium* in humans (Dong et al., 2020) and water reservoirs (Daraei et al., 2021) at 7.6% (95% CI: 6.9–8.5%) and 36% (95% CI: 31.4–40.7%), respectively. The reported prevalence of this parasitic infection is 8% (95% CI: 5–11%) in dogs (Taghipour et al., 2020a), 6% (95% CI: 4–8%) in cats (Taghipour et al., 2021), 17% (95% CI: 13–20%) in rodents (Taghipour et al., 2020b), 16.3% (95% CI: 15–17.6%) in pigs (Chen et al., 2023), and 7.6% (95% CI: 4.8–10.8%) in equines (Li et al., 2022). Our findings revealed that the global prevalence of cryptosporidiosis in camels [13.8% (95% CI: 10.3–18.4%)] is relatively high compared to animals like dogs, cats, and horses, but lower compared to rodents and pigs. It also indicated that camels could serve as a proper source of infection for *Cryptosporidium* infection, highlighting the importance of considering public health and zoonotic infection transmission. Discrepancies in reported prevalences across studies may be attributed to variations in study numbers, sample quality and sizes, animal species, geographical locations, animal husbandry practices, and diagnostic method sensitivity. The sensitivity analysis results showed that excluding any of the studies (datasets) on cryptosporidiosis in camels did not lead to significant statistical changes in the final weighted prevalence. This indicates that no outlier data is present in the studies analyzed in this review that would significantly affect the overall prevalence of *Cryptosporidium* spp. in camels.

Publication-based analysis showed that the highest and lowest infection rates in camels were observed in studies published from 2009 to 2015 and 2002–2008, with rates of 21.7% (95% CI: 15.7–29.1%) and 3% (95% CI: 1.7–5.4%), respectively. Nonetheless, due to variations in study numbers, sample sizes, and locations, a direct comparison regarding publication year and *Cryptosporidium* infection rates in camels remains challenging to precisely ascertain. At the continent level, the highest and lowest prevalence was observed in European [one dataset, 35.7% (95% CI: 29.1–42.9%)] and North American [one dataset, 1.3% (95% CI: 0.2–8.6%)] camels, respectively. In addition, camels in the EUR [one dataset, 35.7% (95% CI: 29.1–42.9%)] and AMR WHO regions [one dataset, 1.3% (95% CI: 0.2–8.6%)] showed the highest and lowest frequency, respectively. Of note, the findings in these sections come from studies using only one dataset, which may not accurately represent the actual prevalence rate in a particular population or area. Therefore, it is important to interpret the results of single dataset analyses carefully. Country-based analyses found the highest occurrence of cryptosporidiosis in camels in Azerbaijan (one dataset), Iraq (six datasets), Nigeria (one dataset), and Ethiopia (two datasets) at 35.7% (95% CI: 29.1–42.9%), 34.2% (95% CI: 20.9–50.9%), 30% (95% CI: 25.4–35.1%), and 26.4% (95% CI: 23.2–29.9%), respectively. However, because of limited geographical coverage and inadequate studies in each country, an accurate understanding of the epidemiology of this parasitic infection remains unattainable. While some groups had limited study numbers, the analysis of groups by sample size showed a distinct correlation with *Cryptosporidium* spp. prevalence in camels: a decrease in sample size (100−300) was associated with higher infection rates [16.6% (95% CI: 10.9–24.5%)], while an increase in sample size (>500) was linked to lower infection rates [2.6% (95% CI: 1.4–4.7%)]. Therefore, to gain a more accurate understanding of *Cryptosporidium* infection across various hosts, a substantial sample size is essential. Articles reporting *Cryptosporidium* spp. prevalence using microscopic (14.8%; 95% CI: 10.1–21.1%) versus serological (13%; 95% CI: 7–22.9%) and molecular (11.8%; 95% CI: 8–17%) methods showed a higher prevalence of this protozoan in camels. However, the discrepancy in the number of studies utilizing diagnostic techniques does not support this conclusion, and the outcomes derived from these analyses should be interpreted with care. The pooled prevalence of *Cryptosporidium* was higher in two-humped camels (14%; 95% CI: 4.8–34.2%) and males (14.1%; 95% CI: 6.8–26.9%) compared to single-humped camels (12.6%; 95% CI: 8.1–19%) and females (12.3%; 95% CI: 4.3–30.4%). Additionally, a correlation was found between lower animal age and higher *Cryptosporidium* infection rates.

In summary, the present study found that eight species of *Cryptosporidium* have been identified in camels: *C. parvum*, *C. andersoni*, *C. bovis*, *C. muris*, *C. ratti*, *C. occultus*, *C. ubiquitum*, and *C. hominis*. Among these, the first three species have pooled prevalence rates of 65.5% [seven datasets (95% CI: 30.5–89.2%)], 66% [four datasets (95% CI: 51.7–77.9%)], and 19.2% [four datasets (95% CI: 4.6–54.1%)], respectively. Whereas, the five remaining species have been reported individually. Of note, species of *Cryptosporidium* identified using microscopic and serological methods in certain studies should be interpreted carefully. Generally, these methods are unreliable for determining species identification, potentially resulting in the false, over- or under-estimation of the final prevalence of species. Moreover, in camels, genotypes IIdA19G1, IIaA15G1R1, If-like-A15G2, IIdA15G1, IIaA15G2R1, IIaA17G2R1, and IIaA18G2R1 from *C. parvum*, genotype IV from *C. ratti*, genotype XIIa from *C. ubiquitum*, and genotype IkA19G1 from *C. hominis* have been found in several countries (Table 3).

A high rate of heterogeneity was identified as publication bias in this study, potentially impacting the outcomes (Thornton and Lee, 2000). This could be due to variations in geographical region, publication year, number of studies, and sample size as shown in Table 2. Other factors not addressed in this review, such as animal health status, sampling methods, sample preservation, and animal-rearing practices, could also contribute to publication bias. Therefore, the findings of this study should be interpreted carefully. Despite the valuable epidemiological data collected in our current study, future research could further illuminate the occurrence and distribution of species and epidemiological trends of *Cryptosporidium* infection in camels worldwide.

## 5 Conclusion

This review and meta-analysis study on *Cryptosporidium* spp. in camels revealed a moderate pooled prevalence (13.8%) of this protozoan infection. The findings indicate that camels can serve as a source of infection for range of *Cryptosporidium* species and genotypes, highlighting the need for preventive measures and medical and veterinary attention in areas with camels. Limitations encountered in this study encompassed: insufficient study diversity, reliance on single study results, inadequate molecular and serological studies in comparison to microscopic studies, etc., all of which could impact the findings. Therefore, it is advised to interpret the results of this study with caution. Extensive and detailed research is required to understand the epidemiology of *Cryptosporidium* and the distribution of its species/genotypes in camels.

The following are the supplementary data related to this article.

Supplementary Fig. 1 Supplementary Fig. 1

Supplementary Fig. 2 Supplementary Fig. 2

Supplementary Fig. 3 Supplementary Fig. 3

Supplementary Fig. 4 Supplementary Fig. 4

Supplementary Fig. 5 Supplementary Fig. 5

Supplementary Fig. 6 Supplementary Fig. 6

Supplementary Fig. 7 Supplementary Fig. 7

Supplementary Fig. 8 Supplementary Fig. 8

Supplementary Fig. 9 Supplementary Fig. 9

Supplementary Fig. 10 Supplementary Fig. 10

Supplementary Fig. 11 Supplementary Fig. 11

Supplementary Table 1 Supplementary Table 1

## CRediT authorship contribution statement

**Farzad Mahdavi:** Methodology, Investigation. **Farajolah Maleki:** Methodology, Investigation. **Mohammad Reza Mohammadi:** Methodology, Investigation. **Ali Asghari:** Writing – review & editing, Writing – original draft, Methodology, Investigation, Conceptualization. **Behnam Mohammadi-Ghalehbin:** Methodology, Investigation.

## Declaration of competing interest

The authors declare no potential conflicts of interest with respect to the research, authorship, and/or publication of this article.

## References

[bb0005] Abd-Al-Aal Z., El-Kabbany A., Tahrani L. (2016). Comparison between two diagnostic methods for detection of *Cryptosporidium* spp. infecting farm animals in Kuwait. Bull Fac Sci Zagazig Univ.

[bb0010] Abdel-Wahab A., Abdel-Maogood S. (2011). Identification of *Cryptosporidium* species infecting camels (Camelus dromedarius) in Egypt. Am. J. Sci..

[bb0015] Abraha A., Urge B., Kemal J., Siyoum T., Tadele M., Muktar Y. (2020). Major microbial and parasitic pathogens causing calf diarrhea of dromedary camel in selected areas of eastern Ethiopia. Livest. Res Results.

[bb0020] Adamu S.G., Madu H.K., Tizhe J.Q. (2012). Prevalence of cryptosporidiosis in dromedary camels (*Camelus dromedarius*) in Yobe state. Nigeria. Sahel J. Vet. Sci..

[bb0025] Al-Megrin W.A.I. (2015). Comparison of ELISA and microscopy for detection of *Cryptosporidium* oocysts in animals. Pak. J. Biol. Sci..

[bb0030] Asghari A., Ebrahimi M., Shamsi L., Sadrebazzaz A., Shams M. (2023). Global molecular prevalence of *Giardia duodenalis* in pigs (*Sus domesticus*): a systematic review and meta-analysis. Heliyon.

[bb0035] Baroudi D., Zhang H., Amer S., Khelef D., Roellig D.M., Wang Y., Feng Y., Xiao L. (2018). Divergent *Cryptosporidium parvum* subtype and *Enterocytozoon bieneusi* genotypes in dromedary camels in Algeria. Parasitol. Res..

[bb0040] Bouragba M., Laatamna A., Cheddad F.E., Baroudi D., Houali K., Hakem A. (2020). Gastrointestinal parasites of dromedary camel (*Camelus dromedarius*) in Algeria. Vet. World.

[bb0045] Cao Y., Cui Z., Zhou Q., Jing B., Xu C., Wang T., Qi M., Zhang L. (2020). Genetic diversity of *Cryptosporidium* in bactrian camels (*Camelus bactrianus*) in Xinjiang, Northwestern China. Pathogens.

[bb0055] Chen Y., Qin H., Wu Y., Xu H., Huang J., Li J., Zhang L. (2023). Global prevalence of *Cryptosporidium* spp. in pigs: a systematic review and meta-analysis. Parasitology.

[bb0060] Daraei H., Oliveri Conti G., Sahlabadi F., Thai V.N., Gholipour S., Turki H., Fakhri Y., Ferrante M., Moradi A., Mousavi Khaneghah A. (2021). Prevalence of *Cryptosporidium* spp. in water: a global systematic review and meta-analysis. Environ. Sci. Pollut. Res..

[bb0065] Dong S., Yang Ya, Wang Y., Yang D., Yang Yu, Shi Y., Li C., Li L., Chen Y., Jiang Q. (2020). Prevalence of *Cryptosporidium* infection in the global population: a systematic review and meta-analysis. Acta Parasitol..

[bb0070] El Hassan E.-A.M., Al-Jabr O.A., El-Bahr S.M. (2020). Prevalence of *Cryptosporidium parvum* in diarrheic camel-calves (*Camelus dromedarius*) in Al-Ahsa, Saudi Arabia. AJVS.

[bb0075] El Kelesh E.A., Abdel-Maogood S.Z., Abdel-Wahab A.M. (2009). Comparative studies on some *Cryptosporidium* species infecting different animals. Egypt. Vet. Med. Soc. Parasitol. J..

[bb0080] El Wathig M., Faye B. (2016). Camel calf diarrhoea in Riyadh region, Saudi Arabia. J. Camel Pract. Res..

[bb0085] El-Alfy E.-S., Abu-Elwafa S., Abbas I., Al-Araby M., Al-Kappany Y., Umeda K., Nishikawa Y. (2019). Molecular screening approach to identify protozoan and trichostrongylid parasites infecting one-humped camels (*Camelus dromedarius*). Acta Trop..

[bb0090] El-Khabaz K.A.S., Abdel-Hakeem S.S., Arfa M.I. (2019). Protozoan and helminthes parasites endorsed by imported camels (*camel dromedaries*) to Egypt. J. Parasit. Dis..

[bb0095] Elmahallawy E.K., Köster P.C., Dashti A., Alghamdi S.Q., Saleh A., Gareh A., Alrashdi B.M., Hernández-Castro C., Bailo B., Lokman M.S. (2023). Molecular detection and characterization of *Cryptosporidium* spp., *Giardia duodenalis*, and *Enterocytozoon bieneusi* infections in dromedary camels (Camelus dromedaries) in Egypt. Front. Vet. Sci..

[bb0100] Elshahawy I., AbouElenien F. (2019). Seroprevalence of *Cryptosporidium* and risks of cryptosporidiosis in residents of Sothern Egypt: a cross-sectional study. Asian Pac J Trop Med.

[bb0105] Fadly R.S. (2015). Studies on blood and enteric protozoans infecting camels at Behera Province, Egypt. Egypt. Vet. Med. Soc. Parasitol. J..

[bb0110] Gaibova H., Iskenderova N., Hajieva N. (2011). Cryptosporidia of the Bactrian camel in Azerbaijan. Proc Azerbaijan Instit Zool.

[bb0115] Gebru M., Zeru F., Hadush A., Girmay S. (2017). Investigation of intestinal parasitic pathogens and risk factors leading to infectious diarrhea complex in camel calves in selected districts of Afar National Regional State, Ethiopia. Ethiop. J. Vet. Sci. Anim. Prod..

[bb0120] Gu Y., Wang X., Zhou C., Li P., Xu Q., Zhao C., Liu W., Xu W. (2016). Investigation on *Cryptosporidium* infections in wild animals in a zoo in Anhui Province. J. Zoo Wildl. Med..

[bb0125] Hasan M.H., Alani A.A.J., Aghwan S.S. (2021). Investigations on gastrointestinal parasites in camels rearing in Nineveh Governorate. Egypt. J. Vet. Sci..

[bb0130] Hussin A.G., Khalaf J.M., Ali H.M. (2015). Detection of intestinal protozoa in camels and their breeders in Najef, Iraq. Res. J. Vet. Pr..

[bb0135] Institute, J.B (2017).

[bb0140] Izadi M., Jonaidi-Jafari N., Saburi A., Eyni H., Rezaiemanesh M., Ranjbar R. (2012). Prevalence, molecular characteristics and risk factors for cryptosporidiosis among Iranian immunocompromised patients. Microbiol. Immunol..

[bb0145] Jawad H.H., Jasim G.A. (2016). Molecular study of *Cryptosporidium* spp. and *Giardia lamblia* which cause diarrhea in camels (*Camillus dromedaries*) in Al-Diwaniyah and Al-Najaf provinces/Iraq. Al-Qadisiyah J. Vet. Med. Sci..

[bb0150] Kareem S.M., Abbas F.H. (2023). Cardiac pathophysiology: real-time PCR detection of *Cryptosporidium parvum* infection in camels. Rev. Latinoam. Hipertens.

[bb0155] Laatamna A.K., Belkessa S., Khalil A., Afidi A., Benmahdjouba K., Belalmi R., Benkrour M., Ghazel Z., Hakem A., Aissi M. (2018). Prevalence of *Cryptosporidium* spp. in farmed animals from steppe and high plateau regions in Algeria. Trop. Biomed..

[bb0160] Li X.-M., Geng H.-L., Wei Y.-J., Yan W.-L., Liu J., Wei X.-Y., Zhang M., Wang X.-Y., Zhang X.-X., Liu G. (2022). Global prevalence and risk factors of *Cryptosporidium* infection in Equus: a systematic review and meta-analysis. Front. Cell. Infect. Microbiol..

[bb0165] Locklear T.R., Videla R., Breuer R.M., Mulon P.-Y., Passmore M., Mochel J.P., Gerhold R., Schaefer J.J., Smith J.S. (2021). Presentation, clinical pathology abnormalities, and identification of gastrointestinal parasites in camels (*Camelus bactrianus* and *Camelus dromedarius*) presenting to two North American veterinary teaching hospitals. A Retrospective Study: 1980–2020. Front. Vet. Sci..

[bb0170] Mahdavi F., Shams M., Sadrebazzaz A., Shamsi L., Omidian M., Asghari A., Hassanipour S., Salemi A.M. (2021). Global prevalence and associated risk factors of diarrheagenic *Giardia duodenalis* in HIV/AIDS patients: a systematic review and meta-analysis. Microb. Pathog..

[bb0175] Mahdi N.K., Ali N.H. (2002). Cryptosporidiosis among animal handlers and their livestock in Basrah, Iraq. East Afr. Med. J..

[bb0180] Maxamhud S., Reghaissia N., Laatamna A., Samari H., Remdani N., Gentekaki E., Tsaousis A.D. (2023). Molecular identification of *Cryptosporidium* spp., and *Giardia duodenalis* in dromedary camels (*Camelus dromedarius*) from the Algerian Sahara. Parasitologia.

[bb0185] Mohammed N.Q., Abd A.H., Ahmed H.S. (2016). Detection of *Cryptosporidium parvum* from feces samples of human and camels by using direct polymerase chain reaction assay technique. Al-Qadisiyah J. Vet. Med. Sci..

[bb0190] Moher D., Shamseer L., Clarke M., Ghersi D., Liberati A., Petticrew M., Shekelle P., Stewart L.A., Group, P.-P (2015). Preferred reporting items for systematic review and meta-analysis protocols (PRISMA-P) 2015 statement. Syst. Rev..

[bb0195] Mosier D.A., Oberst R.D. (2000). Cryptosporidiosis: a global challenge. Ann. N. Y. Acad. Sci..

[bb0200] Nazifi S., Behzadi M.A., Haddadi S.H., Raayat Jahromi A., Mehrshad S., Tamadon A. (2010). Prevalence of *Cryptosporidium* isolated from dromedary camels (*Camelus dromedarius*) in Qeshm Island, Southern Iran. Comp. Clin. Pathol..

[bb0205] Nouri M., Razmyar J., Keyhani P. (1996). A *Cryptosporidium muris*-like parasite in large ruminants in various parts of Iran. Iran J Vet Med..

[bb0210] Ouchene N., Khelifi-Touhami N.A. (2023). Prevalence of *Cryptosporidium* spp. in humans and dromedaries (*Camelus dromedarius*) in Algeria. Vet. stanica.

[bb0215] Radfar M.H., Aminzadeh M. (2012). Prevalence of *Cryptosporidium parvum* in camels in southeast of Iran. Int. J. Infect. Dis..

[bb0220] Razavi S.M., Oryan A., Bahrami S., Mohammadalipour A., Gowhari M. (2009). Prevalence of *Cryptosporidium* infection in camels (*Camelus dromedarius*) in a slaughterhouse in Iran. Trop. Biomed..

[bb0225] Ryan U., Feng Y., Fayer R., Xiao L. (2021). Taxonomy and molecular epidemiology of *Cryptosporidium* and *Giardia*–a 50 year perspective (1971–2021). Int. J. Parasitol..

[bb0230] Saidi R., Mimoune N., Chaibi R., Baazizi R., Abdelouahed K., Khelef D., Kaidi R. (2022).

[bb0235] Salama A., Noaman E.A., Nayel M., El-Kattan A.M., Mahmoud M.A., Dawood A.S., El-Hamid A., Ibrahim S., Elsify A., Zaghawa A. (2023). Prevalence and molecular characterization of four enteric protozoa in dromedary camels (*Camelus Dromedarius*). Alexandria J. Vet. Sci..

[bb0240] Saleh M.A., Mahran O.M. (2007). A preliminary study on cryptosporidiosis in dromedary camels at Shalatin Area, Egypt. Assiut Vet. Med. J..

[bb0245] Savioli L., Smith H., Thompson A. (2006). *Giardia* and *Cryptosporidium* join the ‘neglected diseases initiative’. Trends Parasitol..

[bb0250] Sazmand A., Rasooli A., Nouri M., Hamidinejat H., Hekmatimoghaddam S. (2012). Prevalence of *Cryptosporidium* spp. in camels and involved people in Yazd Province, Iran. Iran. J. Parasitol..

[bb0255] Soltane R., Guyot K., Dei-Cas E., Ayadi A. (2007). Prevalence of *Cryptosporidium* spp.(Eucoccidiorida: Cryptosporiidae) in seven species of farm animals in Tunisia. Parasite.

[bb0260] Taghipour A., Olfatifar M., Bahadory S., Godfrey S.S., Abdoli A., Khatami A., Javanmard E., Shahrivar F. (2020). The global prevalence of *Cryptosporidium* infection in dogs: a systematic review and meta-analysis. Vet. Parasitol..

[bb0265] Taghipour A., Olfatifar M., Foroutan M., Bahadory S., Malih N., Norouzi M. (2020). Global prevalence of *Cryptosporidium* infection in rodents: a systematic review and meta-analysis. Prev. Vet. Med..

[bb0270] Taghipour A., Khazaei S., Ghodsian S., Shajarizadeh M., Olfatifar M., Foroutan M., Eslahi A.V., Tsiami A., Badri M., Karanis P. (2021). Global prevalence of *Cryptosporidium* spp. in cats: a systematic review and meta-analysis. Res. Vet. Sci..

[bb0275] Thornton A., Lee P. (2000). Publication bias in meta-analysis: its causes and consequences. J. Clin. Epidemiol..

[bb0280] Utami W.S., Murhandarwati E.H., Artama W.T., Kusnanto H. (2020). *Cryptosporidium* infection increases the risk for chronic diarrhea among people living with HIV in Southeast Asia: a systematic review and meta-analysis. Asia Pacific J. Public Heal..

[bb0285] Wahba A.A., Radwan I.G.H. (2009). Some studies on protozoal parasites of camels in Egypt. Egypt. J. Comp. Pathol. Clin. Pathol..

[bb0290] Wang X., Zhang Z., Yin W., Zhang Q., Wang R., Duan Z. (2021). Interactions between *Cryptosporidium*, *Enterocytozoon*, *Giardia* and intestinal microbiota in bactrian camels on Qinghai-Tibet Plateau, China. Appl. Sci..

[bb0295] Xie N., Zhong Z., Liu X., Chen W., Li W., Liu J., Deng J., Sun H., Xie Y., Zhuang Y. (2015). Isolation and identification of *Cryptosporidium* genotype and subtype from bactrian camel in Sichuan Province. Chinese Vet. Sci. Shouyi Kexue.

[bb0300] Yakhchali M., Moradi T. (2012). Prevalence of *Cryptosporidium*-like infection in one-humped camels (*Camelus dromedarius*) of northwestern Iran. Parasite J. la Société Française Parasitol..

[bb0305] Zhang Q., Zhang Z., Ai S., Wang X., Zhang R., Duan Z. (2019). *Cryptosporidium* spp., *Enterocytozoon bieneusi*, and *Giardia duodenalis* from animal sources in the Qinghai-Tibetan plateau area (QTPA) in China. Comp. Immunol. Microbiol. Infect. Dis..

[bb0310] Zhang Z., Su D., Meng X., Liang R., Wang W., Li N., Guo Y., Guo A., Li S., Zhao Z. (2022). Cryptosporidiosis outbreak caused by *Cryptosporidium parvum* subtype IIdA20G1 in neonatal calves. Transbound. Emerg. Dis..

