# Supplementary material for: Global epidemiology and species/genotype distribution of Cryptosporidium in camels: A systematic review and meta-analysis
Source: Food Waterborne Parasitol. 2024 Jul 11;36:e00235. doi: 10.1016/j.fawpar.2024.e00235 (PMC11298603; doi:10.1016/j.fawpar.2024.e00235)
Supplement: Supplementary Fig. 10 [file mmc10.docx]

**Supplementary Fig. 10.** Sensitivity analysis and *Cryptosporidium* spp. prevalence in camels.
