# Supplementary material for: Global epidemiology and species/genotype distribution of Cryptosporidium in camels: A systematic review and meta-analysis
Source: Food Waterborne Parasitol. 2024 Jul 11;36:e00235. doi: 10.1016/j.fawpar.2024.e00235 (PMC11298603; doi:10.1016/j.fawpar.2024.e00235)
Supplement: Supplementary Table 1 [file mmc12.docx]

**Supplementary Table 1**

**JBI critical appraisal checklist applied for included studies**

| Author Name/Year | Sample was representative? | Participants appropriately recruited? | Sample size was adequate? | Study subjects and the setting described? | Data analysis conducted | Objective, standard criteria, reliably used? | Appropriate statistical analysis used | Confounding factors/ subgroups/ differences identified and accounted? | Subpopulations identified using objective criteria | Overall quality |
| --- | --- | --- | --- | --- | --- | --- | --- | --- | --- | --- |
| Nouri, 1996 | Yes | Yes | Yes | Yes | Yes | Yes | No | No | No | 6/9 |
| Mahdi, 2002 | Yes | No | Yes | Yes | No | Yes | No | No | No | 4/9 |
| Saleh, 2007 | Yes | No | Yes | No | Yes | No | Yes | No | No | 4/9 |
| Soltane, 2007 | Yes | Yes | No | Yes | No | Yes | No | Yes | No | 5/9 |
| Razavi, 2009 | Yes | Yes | Yes | Yes | Yes | No | Yes | Yes | No | 7/9 |
| Kelesh, 2009 | Yes | Yes | Yes | Yes | Yes | No | Yes | Yes | Yes | 8/9 |
| Wahba, 2009 | Yes | Yes | Yes | Yes | Yes | Yes | Yes | Yes | No | 8/9 |
| Nazifi, 2010 | Yes | Yes | Yes | Yes | Yes | No | Yes | No | Yes | 7/9 |
| Gaibova, 2011 | Yes | Yes | Yes | Yes | Yes | No | Yes | Yes | Yes | 8/9 |
| Abdel-Wahab, 2011 | Yes | No | Yes | Yes | No | Yes | Yes | No | Yes | 6/9 |
| Sazmand, 2012 | Yes | No | Yes | No | Yes | No | Yes | No | No | 4/9 |
| Yakhchali, 2012 | Yes | Yes | No | Yes | No | Yes | No | Yes | No | 5/9 |
| Radfar, 2012 | Yes | Yes | Yes | Yes | Yes | No | Yes | Yes | No | 7/9 |
| Adamu, 2012 | Yes | Yes | Yes | Yes | Yes | No | Yes | No | No | 6/9 |
| AL-Megrin, 2015 | Yes | Yes | Yes | Yes | Yes | No | Yes | Yes | Yes | 8/9 |
| Fadly, 2015 | Yes | Yes | Yes | Yes | Yes | Yes | Yes | Yes | No | 8/9 |
| Xie, 2015 | Yes | Yes | Yes | Yes | Yes | No | Yes | No | Yes | 7/9 |
| Hussin, 2015 | Yes | No | Yes | No | Yes | No | Yes | No | No | 4/9 |
| Jawad, 2016 | Yes | Yes | No | Yes | No | Yes | No | Yes | No | 5/9 |
| Abd-Al-Aal, 2016 | Yes | Yes | Yes | Yes | Yes | No | Yes | Yes | No | 7/9 |
| Ahmed, 2016 | Yes | Yes | Yes | Yes | Yes | No | Yes | No | No | 6/9 |
| Gu, 2016 | Yes | Yes | Yes | Yes | Yes | Yes | Yes | Yes | No | 8/9 |
| El Wathig, 2016 | Yes | Yes | Yes | Yes | Yes | No | Yes | Yes | Yes | 8/9 |
| Gebru, 2017 | Yes | No | Yes | Yes | No | Yes | Yes | No | Yes | 6/9 |
| Laatamna, 2018 | Yes | No | Yes | No | Yes | No | Yes | No | No | 4/9 |
| Baroudi, 2018 | Yes | Yes | No | Yes | No | Yes | No | Yes | No | 5/9 |
| El-Alfy, 2019 | Yes | Yes | Yes | Yes | Yes | No | Yes | Yes | No | 7/9 |
| El-Khabaz, 2019 | Yes | Yes | Yes | Yes | Yes | No | Yes | No | No | 6/9 |
| Zhang, 2019 | Yes | Yes | Yes | Yes | Yes | No | Yes | Yes | Yes | 8/9 |
| Elshahawy, 2019 | Yes | Yes | Yes | Yes | Yes | Yes | Yes | Yes | No | 8/9 |
| Bouragba, 2020 | Yes | Yes | Yes | Yes | Yes | No | Yes | No | Yes | 7/9 |
| El Hassan, 2020 | Yes | Yes | Yes | Yes | Yes | No | Yes | Yes | Yes | 8/9 |
| Cao, 2020 | Yes | No | Yes | Yes | No | Yes | Yes | No | Yes | 6/9 |
| Abraha, 2020 | Yes | No | Yes | No | Yes | No | Yes | No | No | 4/9 |
| Wang, 2021 | Yes | No | Yes | No | Yes | No | Yes | No | No | 4/9 |
| Hasan, 2021 | Yes | Yes | Yes | Yes | Yes | No | Yes | Yes | No | 7/9 |
| Locklea, 2021 | Yes | Yes | Yes | Yes | Yes | No | Yes | No | No | 6/9 |
| Saidi, 2022 | Yes | Yes | Yes | Yes | Yes | No | Yes | Yes | Yes | 8/9 |
| Elmahallawy, 2023 | Yes | Yes | Yes | Yes | Yes | Yes | Yes | Yes | No | 8/9 |
| Kareem, 2023 | Yes | Yes | Yes | Yes | Yes | No | Yes | No | Yes | 7/9 |
| Salama, 2023 | Yes | Yes | Yes | Yes | Yes | No | Yes | Yes | Yes | 8/9 |
| Ouchene, 2023 | Yes | No | Yes | Yes | No | Yes | Yes | No | Yes | 6/9 |
| Maxamhud, 2023 | Yes | No | Yes | No | Yes | No | Yes | No | No | 4/9 |
